# Phenotypic characterization of adenomyosis occurring at the inner and outer myometrium

**DOI:** 10.1371/journal.pone.0189522

**Published:** 2017-12-18

**Authors:** Yohei Kishi, Keiji Shimada, Tomomi Fujii, Tomoko Uchiyama, Chiharu Yoshimoto, Noboru Konishi, Chiho Ohbayashi, Hiroshi Kobayashi

**Affiliations:** 1 Department of Obstetrics and Gynecology, Nara Medical University, Nara, Japan; 2 Department of Obstetrics and Gynecology, Takanohara Central Hospital, Nara, Japan; 3 Department of Diagnostic Pathology, Nara Medical University, Nara, Japan; Michigan State University, UNITED STATES

## Abstract

**Objective:**

To estimate the phenotypic characterization of fibrotic process in adenomyosis occurring at the inner or the outer myometrium.

**Methods:**

Eight cases of adenomyosis occurring at the inner myometrium (Subtype I) and 10 cases of adenomyosis occurring at the outer myometrium (Subtype II), and 10 normal counterparts were used in this study. A immunohistochemical study for smooth muscle cells (SMCs) was performed using cytoskeletal proteins, Type I and III collagen, TGF-β and its signaling molecules.

**Results:**

An increased expression of Type I collagen was observed in the extracellular matrix of adenomyotic foci. In normal uteri, immunostaining of SMC differentiation marker proteins (Desmin, Smoothelin, Myosin heavy chain (MHC)) were absent or only found in low numbers at the inner myometrium, while all of these marker proteins were clearly stained at the outer myometrium. In both types of adenomyotic foci, Desmin, Smoothelin, and MHC commonly showed a negative staining at the adjacent area to the glands. A significant staining of Non-muscle myosin IIB, TGF-β, and phosphorylated TGF-β type I receptors were found only at the SMCs of Subtype II adenomyosis. The Smad3/2 ratio of Subtype II adenomyosis was significantly higher than that of Subtype I.

**Conclusions:**

The inner myometrium of normal uteri was composed of undifferentiated phenotypes of SMCs, while the outer myometrium was composed of terminally differentiated SMCs. Various fibrotic processes have been suggested in the development of uterine adenomyosis. Distinct expression patterns of fibrosis related proteins have been shown to be implicated with differences in the subtypes of adenomyosis.

## Introduction

Uterine adenomyosis is a disease characterized by the presence of endometrial glands and stroma within the uterine myometrium. The ectopic endometrial glands and stromal tissue are microscopically surrounded by hyperplasic and hypertrophic smooth muscle cells. Macroscopically, adenomyotic nodules are recognized as fibrotic, elastic hard-nodules in the uterine myometrium. Uterine adenomyosis has been thought to originate from the inner myometrium, however, recent developments in diagnostic tools including magnetic resonance imaging (MRI) have revealed its diversity in the localization of adenomyosis. We previously reported that there were four subtypes of adenomyosis having different localizations. Among them, Subtype I and Subtype II adenomyosis makes a substantial portion of cases, and each subtype has specific characteristics and patient backgrounds[1]. Subtype I adenomyosis occurs at the inner myometrium and is characterized by higher ages and a greater history of curettage. Subtype II adenomyosis occurs at the outer myometrium and is characterized by a strong relationship with pelvic endometriosis[1]. Approximately 96% (49/51) of Subtype II adenomyotic cases had coexisting endometriosis, while that of Subtype I was only 15% (15/59) [1].

Various hypotheses such as invagination of the endometrium and tissue repair restoration have been accepted for the etiology of adenomyosis, but these concepts are not enough to explain the mechanisms involving the subtypes of adenomyosis occurring at the outer myometrium. The exact etiology of adenomyosis still remains unknown. However, all types of adenomyosis commonly have an aspect of fibrotic disease irrespective of their localization. In this study, we focus on the biological features of the Smooth muscle cells (SMCs) of the myometrium, and its involvement in fibrosis.

Fibrosis is the formation of excess fibrous connective tissues in an organ, and is characterized by an excessive deposition of collagen type I and other extracellular matrix (ECM) proteins [2]. This can affect normal tissue architecture and organ function. The synthesis of collagen type I polypeptides is regulated by two separate pathways: the transforming growth factor-β1 (TGF-β1) activation protein pathway and the Smad signaling pathway [2,3]. Until now, little has been known about ECM collagen production in uterine adenomyosis, although collagen type I is known to be the predominant collagen component in uterine leiomyoma ECM [4].

SMCs are known to retain remarkable plasticity when undergoing reversible phenotype modulation in response to local environmental cues, where the SMCs could form a number of phenotypes by modulating the structural or signal components of the cell [5]. So far, many investigations have been performed relating to phenotypic modulation of SMCs in studies of vascular fibrotic diseases [5]. In response to vascular injuries, the SMCs up-regulate proliferative and synthetic capacity, and play an essential role in vascular repair. However, such a high level of plasticity sometimes causes adverse effects that can contribute to the development of vascular fibrotic diseases like atherosclerosis [5]. Furthermore, a variety of SMC specific proteins have been studied to serve as useful differentiation markers of SMC: Desmin, Smoothelin, and Myosin heavy chains (MHCs) etc. MHCs are known to be the only marker that is expressed exclusively in SMCs, and is a very late marker of SMC differentiation. Desmin is the major muscle-specific intermediate filament protein, and known to be a late differentiation markers for SMCs [6]. The loss of Desmin filaments is well accepted to be linked with the loss of contractile functions and fibrosis [6–9]. Additionally, non-muscle myosin IIB (NM-IIB) is known as one of the most useful definitive “positive” markers of de-differentiated SMCs [5], and appears to be relatively specific to phenotypically modified or embryonic SMCs [5].

TGF-β is well recognized as a key mediator in fibrosis and wound healing. The process is mediated by TGF-β type I and type II receptor (TβRI and TβRII) signaling: By the binding of TGF-β to TβRII, TβRI is recruited and phosphorylated at serine and threonine residues within a juxta membrane region [10], leading to the recruitment and phosphorylation of receptor-regulated Smad (R-Smad) proteins. Activated Smads together with Smad4 are translocated into the nucleus, where they regulate target gene expression. The specificity of signal propagation to Smad molecules is determined by TβRI [11]. Phosphorylation of TβRI at Ser165 located amino-terminal of the GS domain has been reported to be involved in modulation of TGF-β1 signaling [12].

In this study, we use immunohistochemistry to investigate the cytoskeletal proteins of SMCs, ECM collagen, and the TGF-β/Smad signaling molecules to estimate the molecular pathogenesis of fibrosis in adenomyosis occurring at the inner or the outer myometrium.

## Materials and methods

### Categorization of adenomyosis

**Fig 1:**
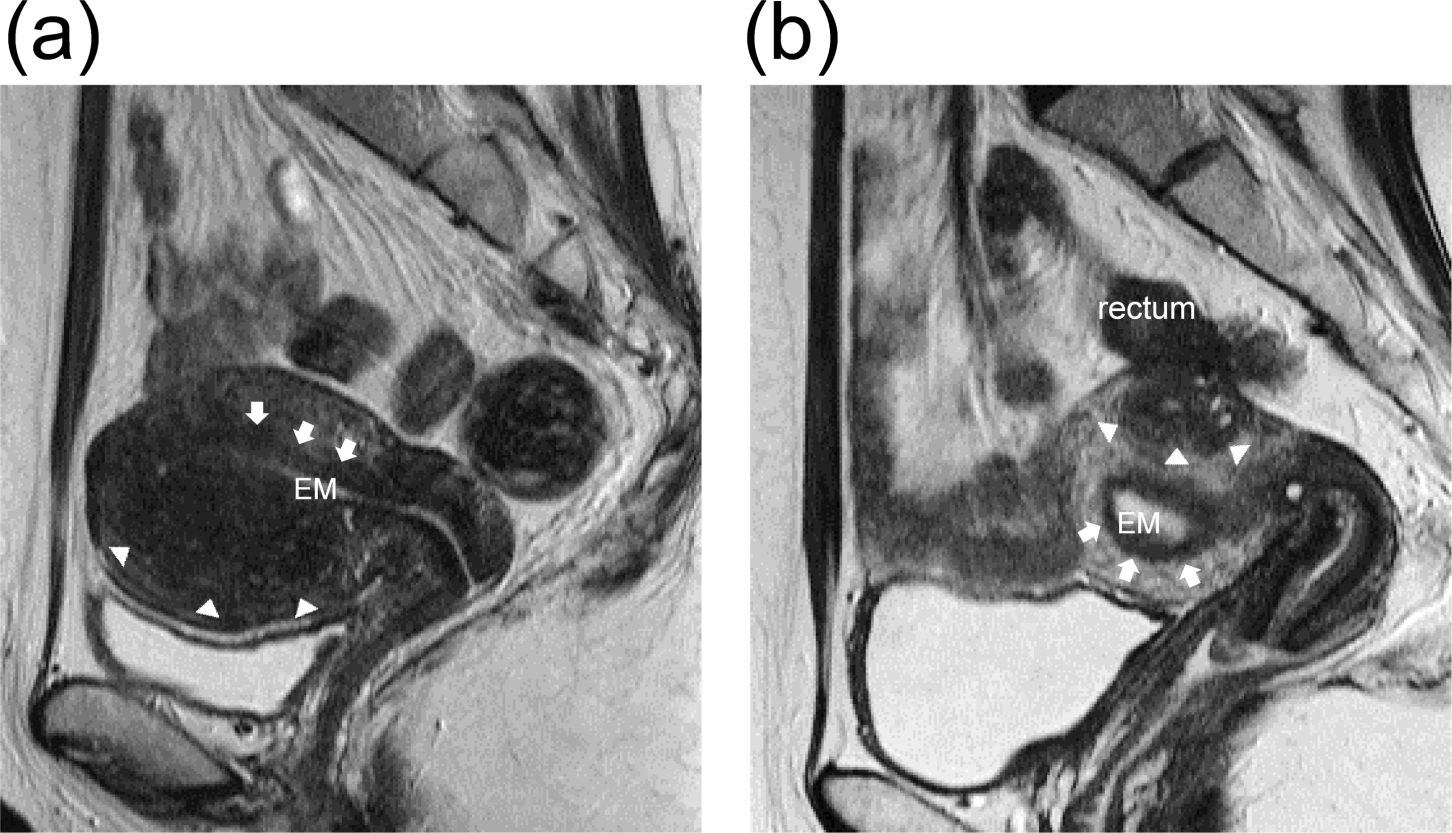
(a) **Subtype I adenomyosis**. T2-weighted magnetic resonance image (sagittal section) of a uterus with Subtype I adenomyosis. The endometrium (EM) is seen as a T2 high intensity band at the inner most part of the uterus. Adenomyosis resides at the inner myometrium with a direct connection to the junctional zone (JZ). The JZ is a T2 low intensity band surrounding the endometrium (arrows). Adenomyotic foci are seen as a T2 low intensity areas with small high intensity spots in the myometrium. The border between the adenomyotic foci and the outer myometrium are indicated by arrow heads. The outer myometrium is preserved and intact. This image was taken for a 39-year old multiparous woman. **(b) Subtype II adenomyosis.** T2-weighted magnetic resonance image (sagittal section) of a uterus with Subtype II adenomyosis. Adenomyosis resides in the posterior outer myometrium without affecting the JZ (arrows). The border between the adenomyotic foci and the surrounding myometrium is indicated by arrows. The healthy muscle structures are seen in between the adenomyosis and the JZ. Utero-rectal adhesion caused by pelvic endometriosis is suspected. This image was taken for a 30-year old nulliparous woman.

We defined the Subtype I adenomyosis as that seen at the inner myometrium affecting the junctional zone (JZ), where healthy myometrium is recognizable on the outer side of the foci using MRI (Fig 1A). Furthermore, we defined Subtype II adenomyosis as that seen focally at the outer myometrium without affecting the JZ, where healthy myometrium is recognizable at the inner side of the foci using MRI (Fig 1B). The definition of each Subtype of adenomyosis is the same as that in a previous study [1].

### Patients

This study was approved by our institutional ethics committee (Study Identification Number: No. 2013005), where written informed consent was obtained from each patient. We retrospectively analyzed paraffin-embedded specimens immunohistochemically. In total 18 cases of adenomyosis were obtained from hysterectomy or adenomyomectomy specimens at Takanohara Central Hospital, Nara, Japan (from April 2007 to December 2012). These 18 adenomyotic cases consisted of 8 Subtype I cases and 10 Subtype II cases. In order to highlight the difference between these two subtypes of adenomyosis, we selected relatively small sized adenomyosis cases for each subtype that was clearly distinguished by MRI. All adenomyotic cases were diagnosed by preoperative MRI and histologically. The basic criteria used for the definition of adenomyosis during MRI were: 1) a myometrial mass with indistinct margins of primarily low intensity with all sequences; or 2) diffuse or local widening of the junctional zone (JZ) on T2-weighted images (>12 mm) [13–15]. The clinical symptoms included menorrhagia and/or pain symptoms that were found in all adenomyotic cases. None of the women with adenomyosis had any history of hormonal treatment within 12 months prior to surgery. None of the women with adenomyosis had any other myometrial disorders including uterine myoma in MRI. To obtain pure specimens of Subtype II adenomyosis, we carefully dissociate the adenomyotic nodules from deep endometriosis nodules. Five hysterectomized Subtype I adenomyotic patients were multiparous women (Table 1). Ten control samples were obtained from hysterectomy specimens, where early cervical neoplasm (CIN) of the uterine cervix was the only indication for the operation. None of the control women had a history of curettage, cesarean sections, uterine surgery or a history of hormonal treatment. Patient demographics are shown in Table 1.

**Table 1:** Patient demographics and clinical characteristics.

	Control	Subtype I adenomyosis	Subtype II adenomyosis	*P* value
Age (average, range)	36 (25–41)	42 (37–46)	33 (20–45)	<0.05
Gravidity (average, range)	2.5 (0–4)	1.9 (0–3)	0.2 (0–2)	<0.05
Parity (average, range)	1.9(0–3)	1.6 (0–3)	0 (0)	<0.05
Hysterectomy	10/10	5/8	0/10	NA
Coexisting endometriosis (n/n)	1/10	1/8	10/10	<0.05

Control: control group, The P values were obtained from a one-way analysis of variance.

### Immunohistochemistry

All surgical specimens were fixed with 10% formalin and embedded in paraffin. After the histological diagnosis on hematoxylin and eosin stained slides, we cut the same paraffin embedded blocks into 4μm sections for immunohistochemistry. The sections were deparafinized in xylene, dehydrated in a graded ethanol series, and rinsed with distilled water, followed by autoclave antigen retrieval at 121°C for 15 min. The immunohistochemical antibodies for each method are shown in Table 2.

**Table 2:** Source, dilutions, antigen retrieval, and incubation methods for the primary antibodies used in immunostaining.

Antigen	Clone /Product ID	Source	Dilution	Antigen retrieval	Incubation time
α-SMA	1A4	Nichirei	Pre-diluted	Citrate PH6.0	1h
Desmin	D33	Nichirei	Pre-diluted	Citrate PH6.0	1h
Smoothelin	R4A	SANTA CRUZ	1:250	Citrate PH6.0	overnight
MHC	SMMS-1	Abcam	1:100	Citrate PH6.0	overnight
NM-IIB	3H2	Abcam	1:200	Citrate PH6.0	1h
Type I COL	EPR7785	Abcam	1:250	Citrate PH6.0	1h
Type III COL	COL3	Abcam	1:250	Citrate PH6.0	1h
TGF-β1	SC-146	SANTA CRUZ	1:100	EDTA	1h
TβRI (S-165)	OAAI00768	AVIVA SYSTEMS BIOLOGY	1:100	Citrate PH6.0	overnight
Smad 2	ab63576	Abcam	1:2000	Citrate PH6.0	1h
Smad 3	EP568Y	Abcam	1:3000	Citrate PH6.0	overnight

### Semi-quantitative digital image analysis of Collagen I and III staining

The degree of Collagen I and III staining was evaluated semi-quantitatively by measuring the maximum thickness of the stained collagen fibrils between the muscle fibers/bundles in 10 different high-power fields for each slide. The average thickness of the collagen bands was compared between the groups. The semi-quantitative digital image analysis of immunohistochemical staining was performed with the use of ImageJ digital image analysis public domain software (developed at the National Institute of Health, US). The sections were observed at 100X, and the thickness of the collagen bands were measured using the digital distance measurement program.

### Immunohistochemical scoring for Smad2 and Smad3 staining

Slides were evaluated independently by two gynecologists and two skilled pathologists. Five different view fields for each slide were evaluated. The scoring system of Smad 2 and Smad 3 were defined as follows: Total score (0–12) = cytoplasmic stain (percentage score (0–3) + intensity score (0–3)) + nuclear stain (percentage score (0–3) + intensity score (0–3).

### Statistical analysis

The one-way analysis of variance (ANOVA) with a Tukey post hoc pairwise comparison was used for the comparison of more than three groups. Nonparametric continuous variables between two groups were compared with the Mann-Whitney U test. P values of < .05 were considered statistically significant.

## Results

Table 1 lists the baseline patient demographics and clinical characteristics. There was a significant difference in age between groups by one-way ANOVA (F (2,25) = 7.06, p<0.05). A Tukey post hoc test revealed that the age of control and Subtype I groups was significantly higher than Subtype II group (p <0.05, p<0.01). There was no significant difference in age between the control and Subtype I group. There was a significant difference in gravidity and parity between groups by one-way ANOVA (gravidity; F (2,22) = 12.26, p<0.05, parity; F (2,25) = 23.40, p<0.05). A Tukey post hoc test revealed that the gravidity and parity of control and Subtype I groups was significantly higher than Subtype II group (P<0.01). There was no statistically significant difference in gravidity and parity between the control and Subtype I groups. There were no hysterectomized patients in Subtype II, while five multiparous patients were hysterectomized in Subtype I. There was a significant difference in the frequency of associated endometriosis between groups by one-way ANOVA (gravidity; F (2,25) = 35.78, p<0.05). A Tukey post hoc test revealed that Subtype II group had associated pelvic endometriosis in significantly higher frequency than the control and Subtype I groups (P<0.01). There was no statistically significant difference in the frequency of associated endometriosis between control and Subtype I groups.

### Type I collagen, Type III collagen immunostainings in control and adenomyosis cases

**Fig 2:**
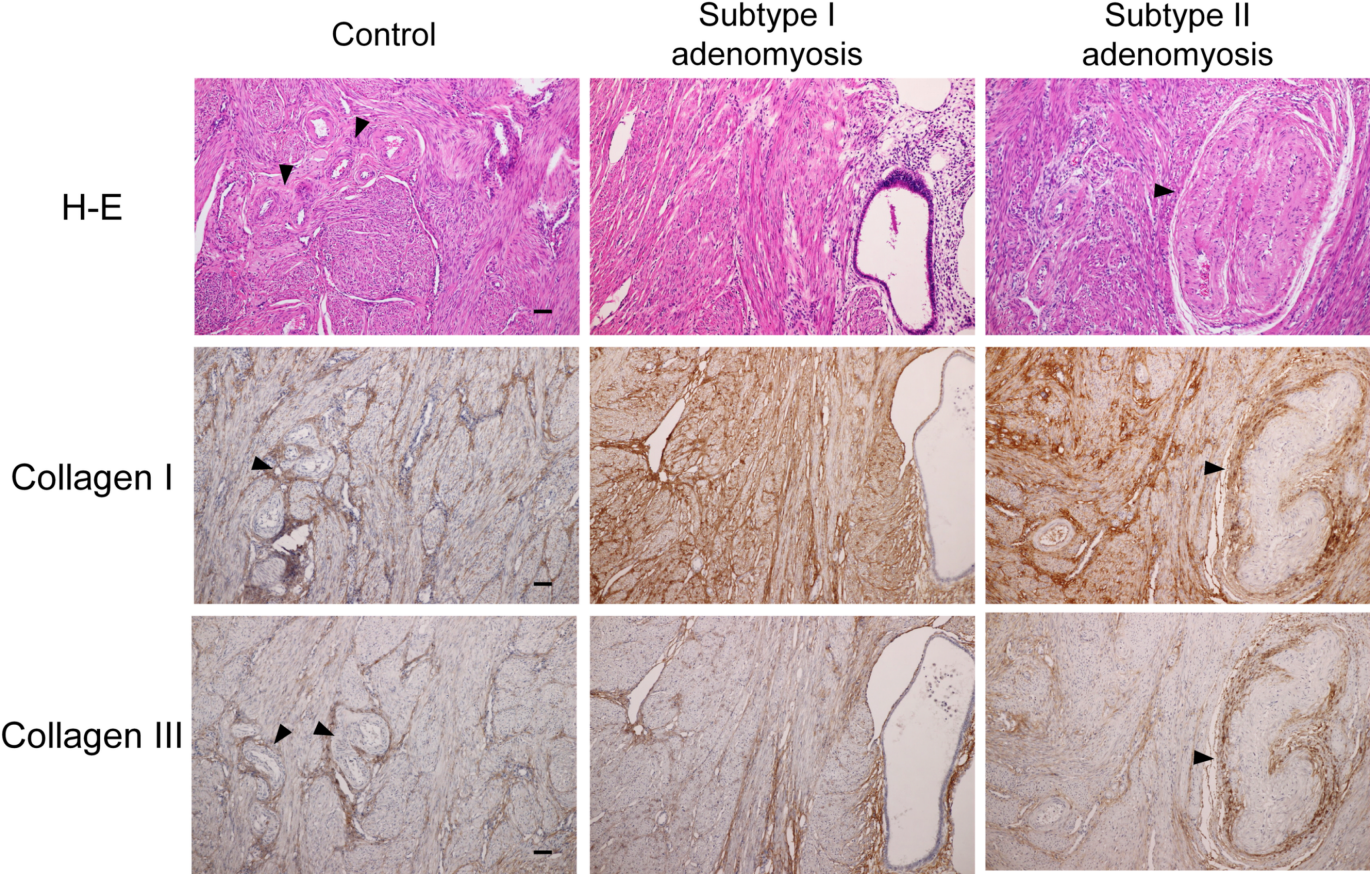
Type I collagen and Type III collagen immunostainings for control, Subtype I, and Subtype II adenomyotic cases. The type I collagen staining bands for adenomyotic cases were thicker than those of the control uteri, and were seen with more fine muscle bundles. Arrowheads indicate vascular walls. Original magnification: X100. Scale bar = 50μm.

**Fig 3:**
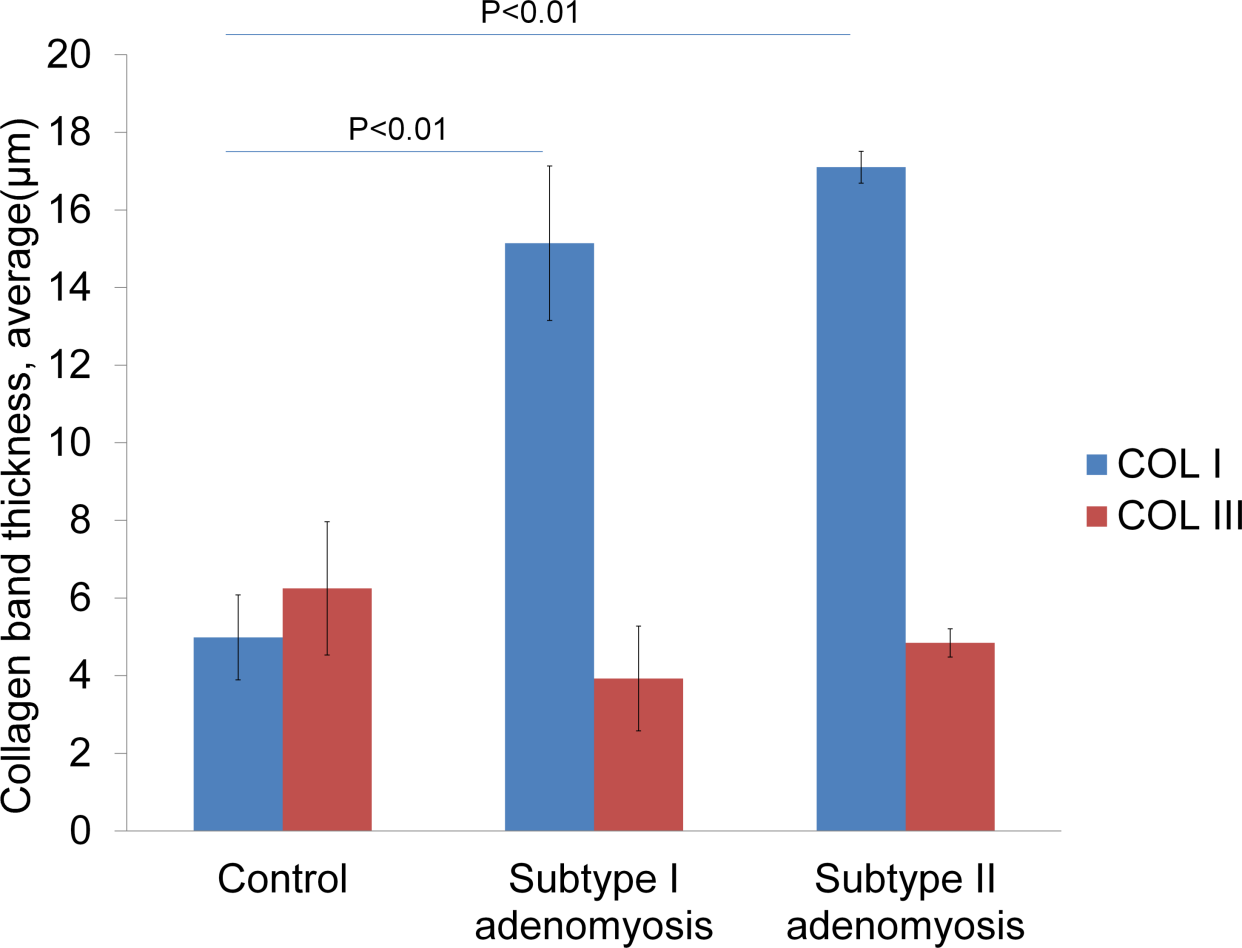
Type I and Type III collagen semi-quantitative comparison graphs. The *P* values were obtained from one-way analysis of variance.

In control uteri, thin collagen staining was seen in the vascular wall smooth muscle fibers (Fig 2). In the semi-quantitative measurement, there was no significant difference between the type I and III collagen amounts in the control uteri (Fig 3). In contrast, both types of adenomyosis showed prominent type I collagen staining compared to type III collagen staining. The type I collagen staining bands for adenomyotic cases were thicker than those of the control uteri, and were seen between more fine muscle bundles. The semi-quantitative comparison data of type I and III collagen for both types of adenomyosis is shown in Fig 3. There was a significant difference in type I collagen semi-quantitative scores between groups by one-way ANOVA (F (2,25) = 30.05, p<0.05). A Tukey post hoc test revealed that the type I collagen semi-quantitative scores of both Subtypes of the adenomyosis groups were significantly higher than the control group (P<0.01). There was no statistically significant difference in type I collagen semi-quantitative scores between Subtype I and Subtype II groups. There was also no significant difference in type III collagen semi-quantitative scores between groups by one-way ANOVA (F (2,25) = 0.284).

### α-SMA, Desmin, Smoothelin, and MHC immunostainings in control uteri

**Fig 4:**
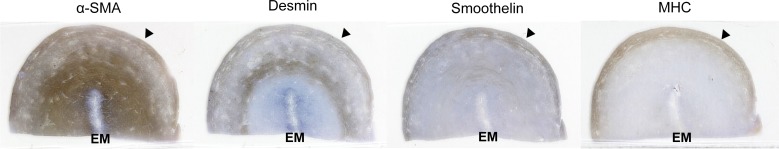
Lupe images of α-SMA, Desmin, Smoothelin and MHC immunostainings of the control uterus. Consecutive coronal sections through the entire myometrium of the uterine fundus area. These specimens were obtained from a hysterectomized 39-year old multiparous woman. Original magnification: X3. EM: endometrium. Arrowheads indicate the uterine serosal surface.

**Fig 5:**
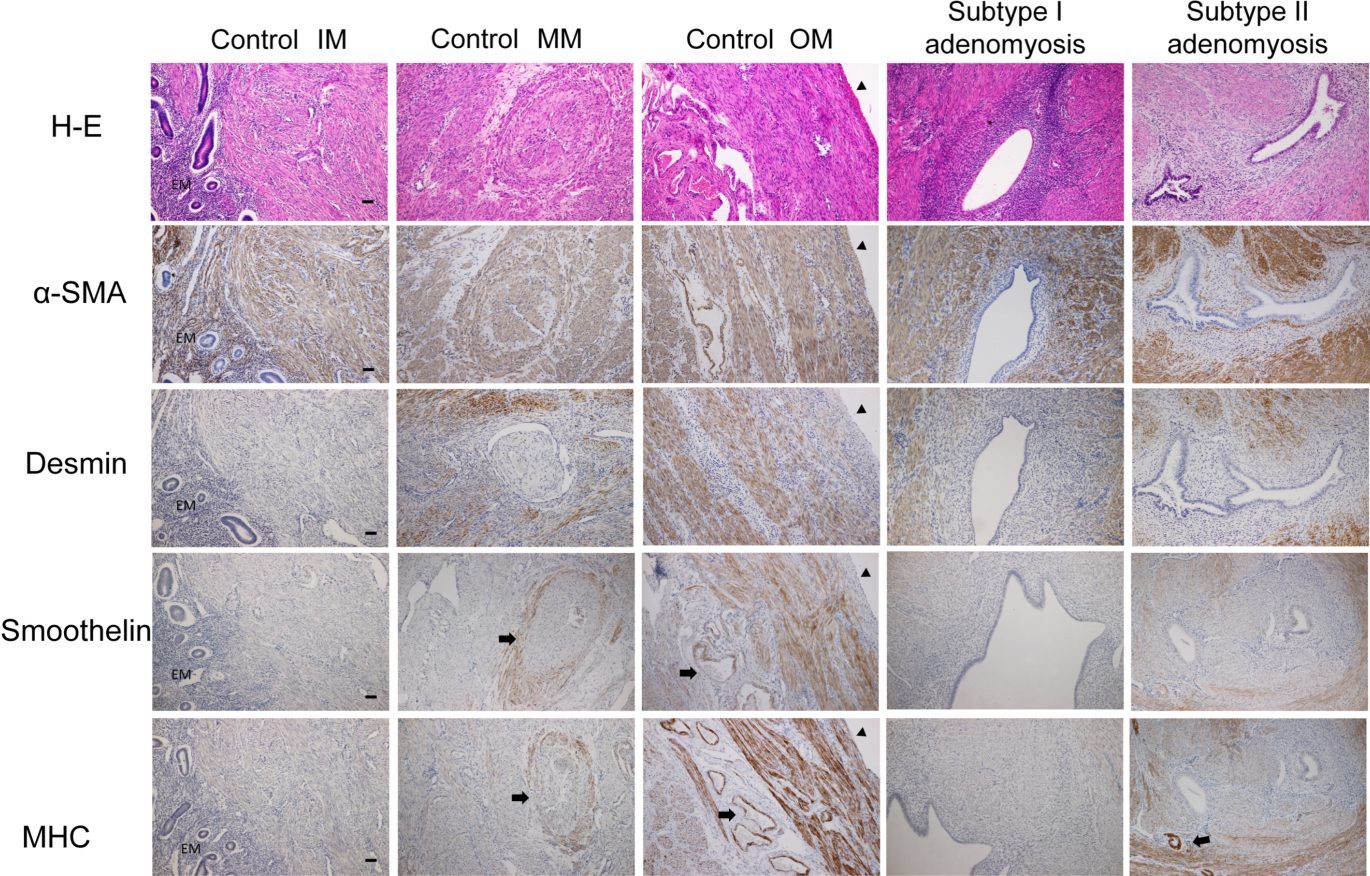
α-SMA, Desmin, Smoothelin, and MHC immunostainings for control, Subtype I, and II adenomyotic cases. (Control IM, MM, OM: inner, middle, and outer myometrium of control uteri.) The whole myometrium for control and adenomyotic uteri were uniformly stained by α-SMA. Desmin, Smoothelin, and MHC staining was depleted or decreased at the inner myometrium of the control uteri. The vascular walls are stained by Smoothelin and MHC as an internal positive control (arrows). In adenomyotic cases, Desmin, Smoothelin and MHC negative SMCs are mainly observed at the proximal of the glands. EM: endometrium. Arrowheads indicate the uterine serosal surface, while arrows indicate vascular walls. Original magnification: X100. Scale bar = 50μm.

Fig 4 shows a typical immunostaining pattern for normal uteri. The whole myometrium was uniformly stained by α-SMA. In contrast, the other cytoskeletal proteins were not stained uniformly. SMCs of the most outer myometrium were clearly stained by Desmin, Smoothelin, and MHC. However, the rest of the myometrium was negative, or weekly positive for Desmin, Smoothelin, and MHC staining. The vascular walls of the middle myometrium layer were stained by Smoothelin and MHC as an internal positive control (Fig 5). A magnified view of each layer is shown in Fig 5.

### α-SMA, Desmin, Smoothelin, and MHC immunostainings in adenomyosis cases

The results are shown in Fig 5, where the SMCs of the adenomyotic foci were uniformly stained by α-SMA. Meanwhile, Desmin staining of these foci were not uniform. A common characteristic of Desmin staining among Subtype I and II adenomyotic foci we found was that Desmin negative SMCs were predominant in the proximity of the glands. The distribution of Desmin negative SMCs in adenomyotic foci were akin to “miniature uterus like”. Smoothelin and MHC had similar staining patterns as compared to Desmin. The distribution of Smoothelin and MHC negative SMCs of adenomyotic foci were also similar to that of Desmin staining in terms of having a tendency to be negative in the proximity of the glands.

### Non-muscle myosin IIB, TGF-β and its signaling molecules in control and adenomyotic cases

**Fig 6:**
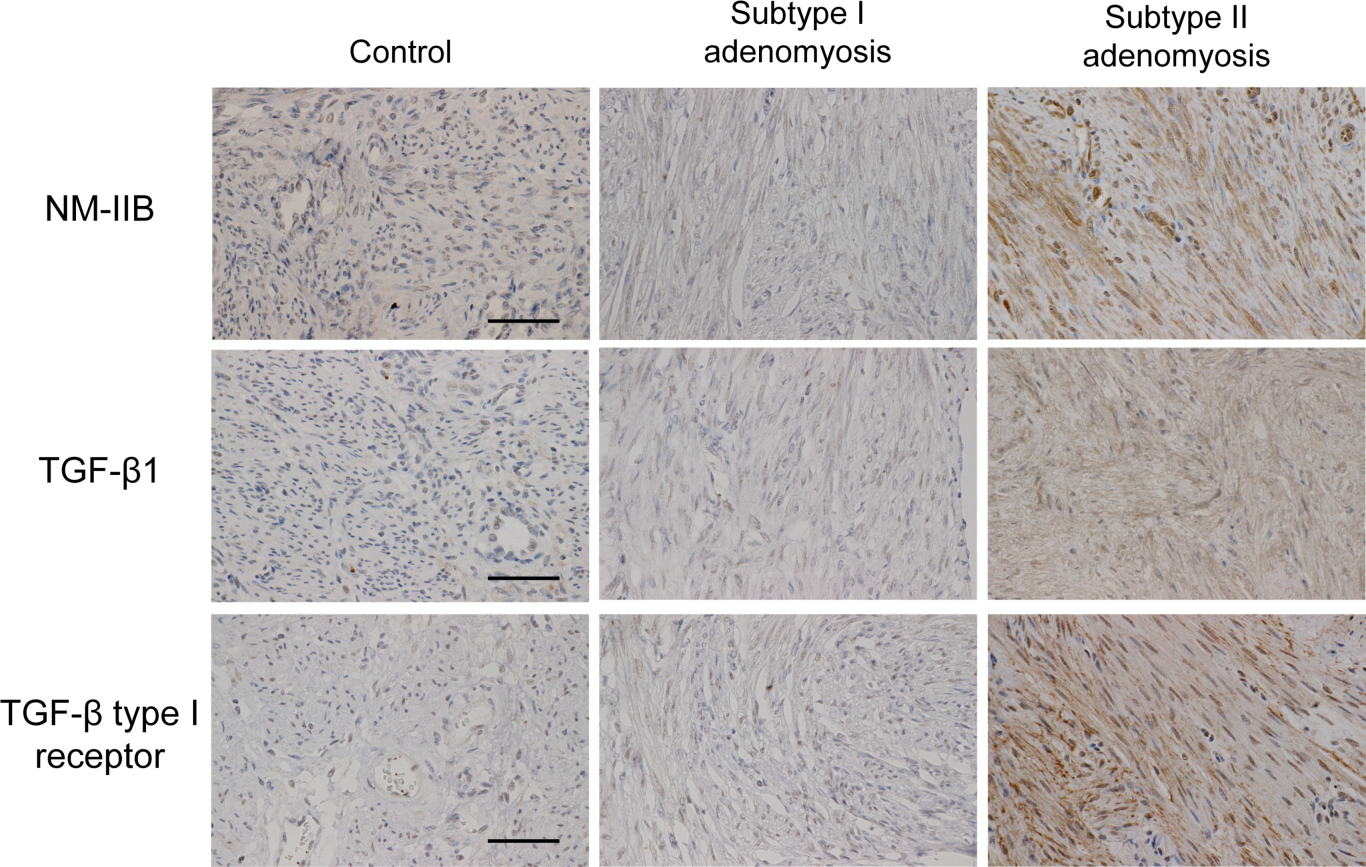
NM-IIB, TGF-β1, TGF-β type I receptor (phosphorylated S-165) immunostainings for control, Subtype I and II adenomyotic cases. A strong cytoplasmic staining of NM-IIB is seen only for Subtype II cases. A clear membrane/cytoplasmic staining of TGF-β and TGF-β type I receptors is seen only for Subtype II cases. Original magnification: X400. Scale bar = 50μm.

The results are shown in Fig 6. In control and Subtype I adenomyotic cases, the expression of NM-IIB was not found at the SMCs. In contrast, a strong and wide range of NM-IIB cytoplasmic staining of the SMCs were observed in all Subtype II cases.

TGF-β1staining was not observed at the SMCs for control and Subtype I cases. However, a wide range of SMC membrane/cytoplasmic staining for TGF-β1was observed in 8/10 cases of Subtype II adenomyosis. Also, SMC staining for phosphorylated TGF-β type I receptor was not seen at the control and Subtype I adenomyotic cases. Meanwhile, 8/10 cases of Subtype II adenomyosis showed a clear membrane/cytoplasmic SMC staining for the phosphorylated TGF-β type I receptor.

**Fig 7:**
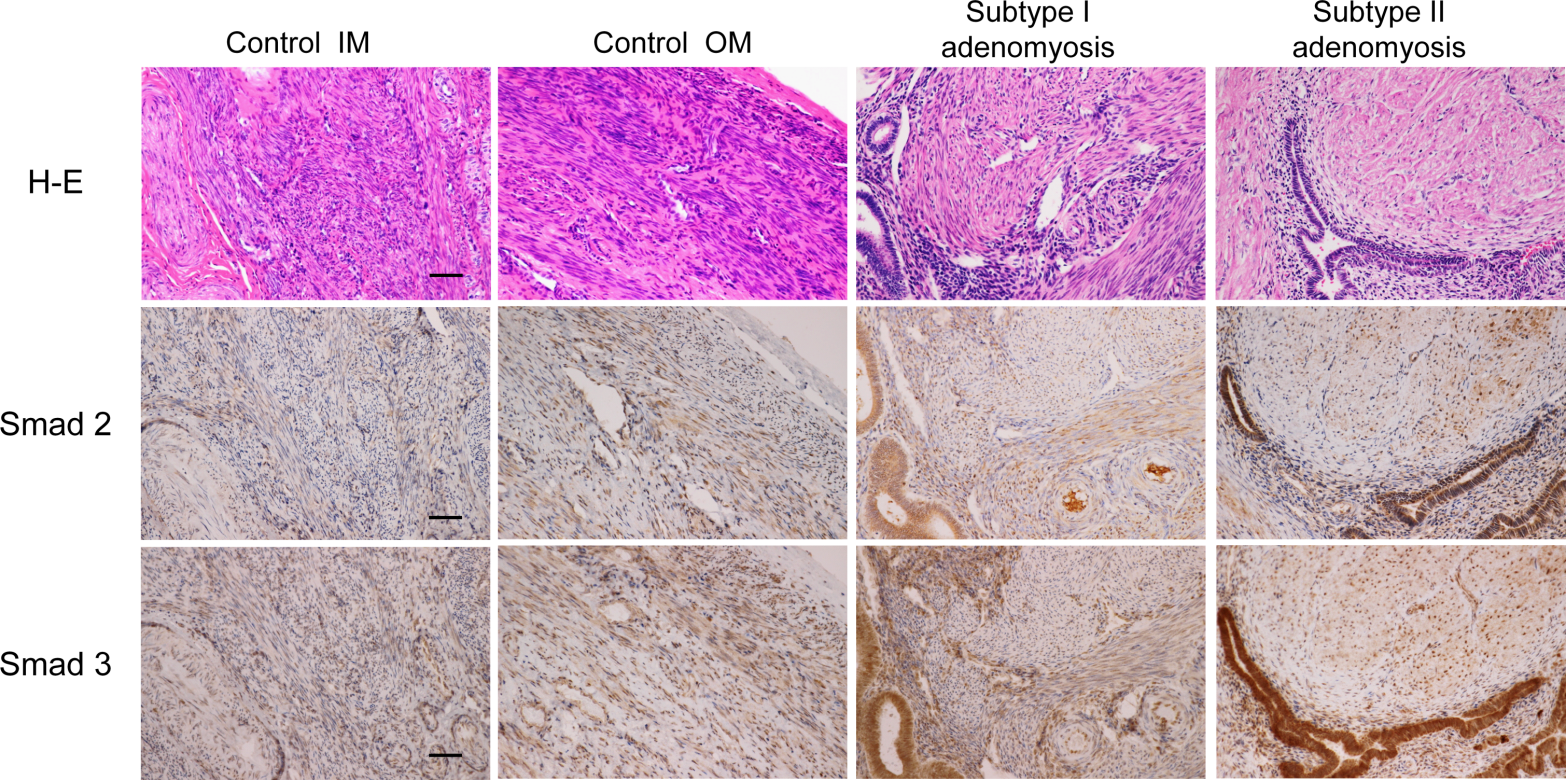
Smad2 and Smad3 immunostainings for control, Subtype I and II adenomyotic cases. Control IM, OM: inner and outer myometrium of control uteri. Original magnification: X200. Scale bar = 50μm.

**Fig 8:**
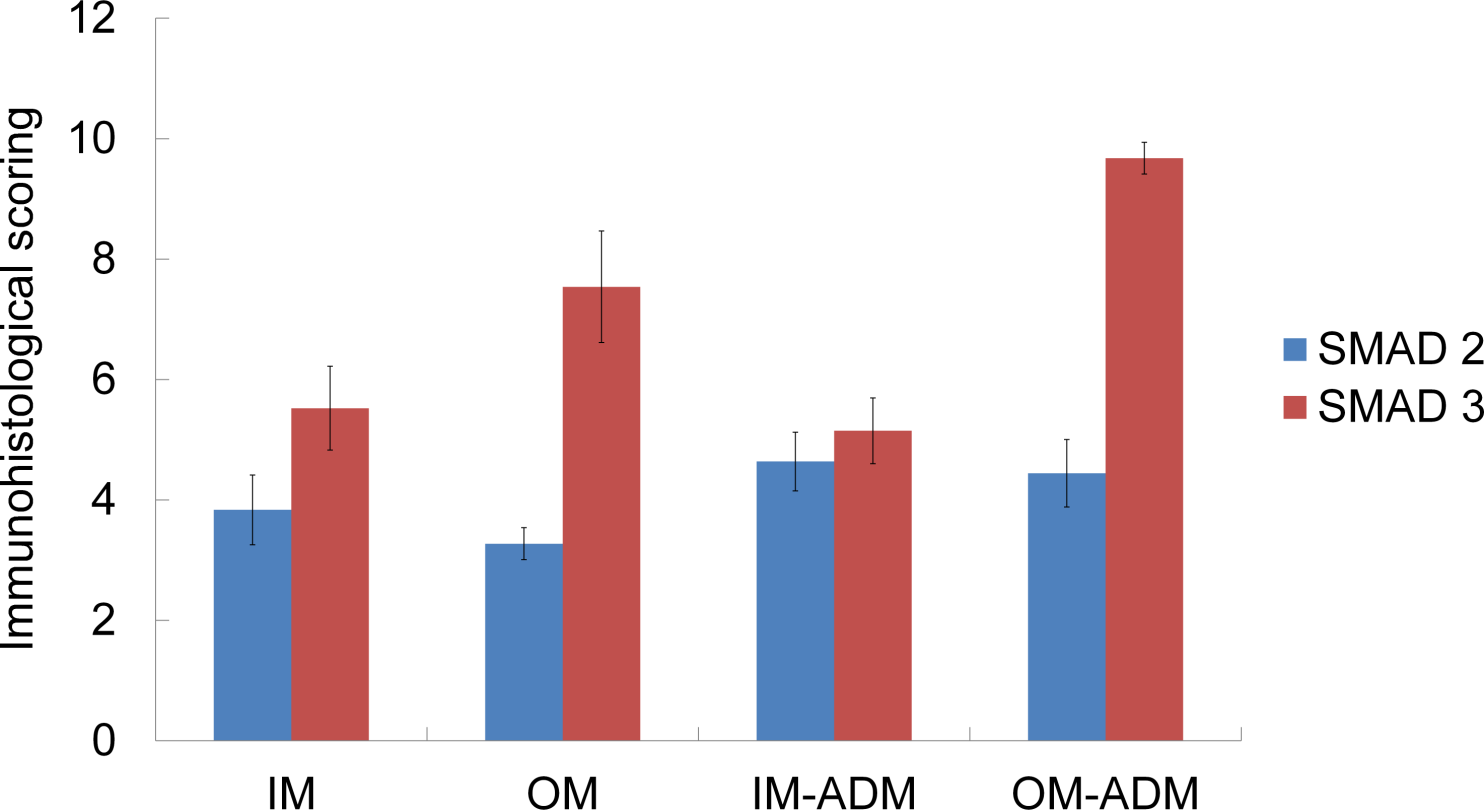
Smad2 and Smad3 immunohistological scorings of control uteri, Subtype I and II adenomyotic foci. IM: inner myometrium of control uteri. OM: outer myometrium of control uteri.

**Fig 9:**
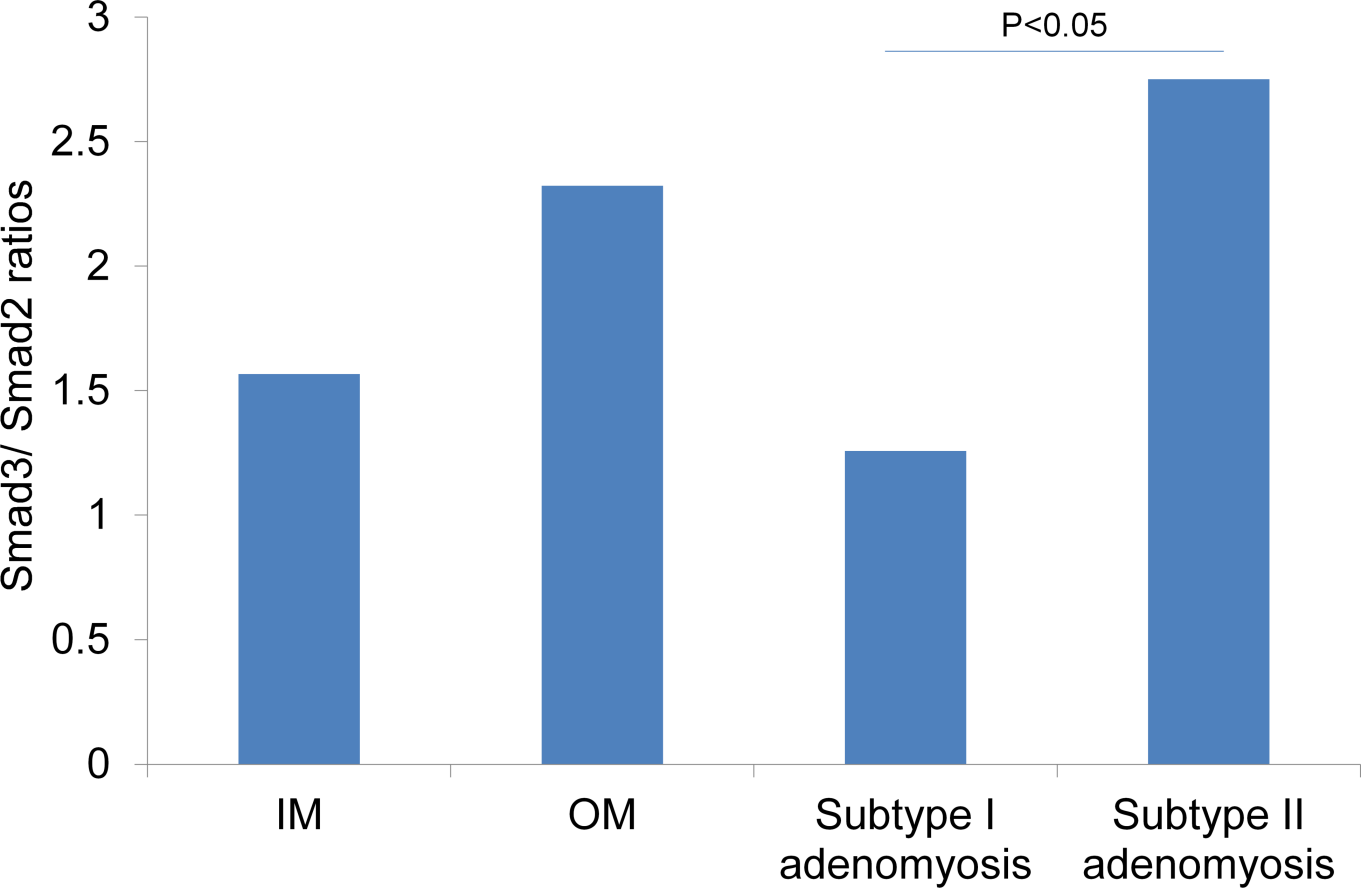
Smad3/Smad2 ratios of control uteri, Subtype I and II adenomyotic foci. IM: inner myometrium of control uteri. OM: outer myometrium of control uteri. The *P* value was obtained from the Mann-Whitney U test.

We further evaluated the total Smad2 and total Smad3 staining of control and each type of adenomyosis. Immunostainings for the total Smad2 and total Smad3 are shown in Fig 7. The immunohistological scorings and Smad3/2 ratios are shown in Figs 8 and 9. There was a statistically significant difference in Smad3/2 ratios between groups by one-way ANOVA (F (3,34) = 3.05, p<0.05). Although a Tukey post hoc test did not show a significant difference between these four groups, the Smad3/2 ratio of Subtype II adenomyosis was significantly higher than that of Subtype I group in Mann-Whitney U Test (p< 0.05).

## Discussion

In this study, we used immunohistochemistry to visualize cytoskeletal components in individual SMCs and their ECM proteins of the uterine myometrium. We confirmed increased type I collagen expression in adenomyotic foci as previously reported [16]. Adenomyosis could be a disease having an aspect of fibrosis irrespective of its localization.

At the beginning of the study, we studied the characteristics of SMCs of the normal myometrium to know the background of the pathogenesis of adenomyosis. As a result, we found that the normal myometrium was composed of different phenotypes of SMCs. The outer myometrium was composed of terminally differentiated SMCs having positive staining for late differentiation marker cytoskeletal proteins. While on the other hand, the inner myometrium was composed of non-differentiated SMCs having the potential to enhance their cell proliferation and ECM production. Especially, the inner most myometrium was negative for all the late differentiation markers that we studied, and was considered to be a sort of Myofibroblast. Thus, it is important to note that each subtype of adenomyosis arises from different environments, and actually each has a specific clinical characteristic.

Subtype I adenomyosis is the classic concept of adenomyosis that could be explained by a direct invasion of the endometrium. Surgical curettage and multiparity are suspected to be the predisposing factors for this type of adenomyosis [1]. It has been considered that traumatic damage of the endometrium-myometrium interface by surgical interventions would allow the endometrium to invade the myometrium. Barrier damage may also occur during normal pregnancy by repeated trophoblastic invasion. For this reason, “age” could be an important factor to be predisposed to these potential factors. However, not all women with this type of adenomyosis have a history of pregnancy or curettage (7/8 (87%) had a history of pregnancy, and 2/8 (25%) had a history of curettage). There could be another formation process in Subtype I adenomyosis. Currently, “tissue injury and repair theory” is widely accepted as a pathogenic theory of adenomyosis [17]. This theory explains how physiological peristalsis causes micro trauma of SMCs which links to reactive inflammatory response and tissue repair. It is thus, essential to understand the functional aspects of the inner myometrium. The inner myometrium plays a role in passive sperm transport and detachment of endometrial cells by its regulated peristaltic contraction through the menstrual cycle.

From our immunohistochemical results, the SMCs of the inner myometrium are supposed to have the potential to increase proliferation rates and synthetic capacity in response to physiological tissue damage. Above all, Desmin negative SMCs are well accepted to be involved in SMC hypertrophy, fibrosis, and muscle inflammation from injury[6–9]; the loss of Desmin is linked to a loss of interfibrillar mechanotransduction, which leads to muscle injury [9]. In this study, Subtype I adenomyosis cases did not show a significant modification of TGF-β1 and Smad3/2 ratios compared to control cases. The TGF-β/Smad signaling pathway is recognized as a master regulator of fibrosis, however there are some different independent fibrotic pathways: mechanical stress and mechanical stiffness independent of TGF-β signaling, induced myofibroblast activation and ECM formation by collagen synthesis [18–21]. A recent study reported that chronic tissue trauma also stimulates the expression of collagen I, but not TGF-β in the inner myometrium [22]. This is consistent with our results. At this stage, we speculate that non-inflammatory pathway like mechanical stress or chronic tissue trauma may be involved in the fibrosis process in Subtype I adenomyosis. Our results suggest that the lower differentiated nature of the inner myometrium, and physiological role of it could be an occurrence factors of fibrosis in Subtype I adenomyosis.

In contrast, Subtype II adenomyosis is formed at the outer myometrium which is composed of highly differentiated SMCs. Because of its localization, it is also difficult to explain by direct invasion of the endometrium. Since the patient background is different from that of Subtype I adenomyosis, there is thought to be a different formation process in Subtype II adenomyosis. We focused on the strong relationship between Subtype II adenomyosis and pelvic endometriosis. The vast majority of Subtype II adenomyosis patients have pelvic endometriosis, which are localized at the outer myometrium where they have a direct connection with peritoneum. In our study, all the Subtype II adenomyosis cases (10/10) had associated rectovaginal endometriosis. The rectovaginal endometriosis is one of the most severe forms of pelvic endometriosis, and often contacts with the serosal surface of the uterine posterior wall. Subtype II adenomyosis cases in this study are supposed to have arisen under the direct and indirect influence from pelvic endometriosis.

In this study, we found Subtype II adenomyosis specific positive staining of NM-IIB, which is a definitive “positive” marker of de-differentiated SMCs that is relatively specific for phenotypically modified or embryonic SMCs [5]. These NM-IIB positive SMCs are considered to have the potential to enhance cell proliferation, ECM production, and to be involved in fibrotic process. We consider that one possible origin of these NM-IIB positive SMCs is terminally differentiated SMCs of the original outer myometrium. Recently, it has been revealed that the TGF-β/Smad system can stimulate de-differentiation of SMCs in high Smad3/Smad2 ratio circumstances [23–25]. Peritoneum is reported to be a source of TGF-β1, and enhanced around endometriosis lesions [26]. In our study cases, TGF-β1/ TGF-β1 receptor expressions with a higher Smad3/Smad2 ratio was observed in Subtype II adenomyosis cases, while Subtype I cases showed no significant modification of TGF-β1signaling proteins. We suspect that TGF-β1 induced de-differentiation of the outer myometrial SMCs may be involved in the fibrotic process of Subtype II adenomyosis.

However, at this stage our results can suggest only a possible process of de-differentiation of SMCs under the influence of pelvic endometriosis. There could still be some possibilities relating to the origin of these NM-IIB positive SMCs: de-differentiated from terminally differentiated SMCs of the outer myometrium or originating from local fibroblasts or mesenchymal cells of bone marrow origin etc.

The etiology of adenomyosis is quite difficult to figure out, however, we believe that our study could provide a new viewpoint that adenomyosis might have some different fibrotic process according to their localization.

We should also mention the limitations of this study. Our results were limited by this study’s retrospective nature, and we only used immunohistological methods. Secondly, the small number of the samples should be taken into consideration. We selected only early stage cases for each subtype of adenomyosis that was clearly distinguished by MRI. By this, however we could compare pure cases of each subtype of adenomyosis. A larger confirmation study is preferable to generalize the interpretation of the results. Furthermore, our study only focused on the characteristics and modification of SMCs in the fibrotic process of adenomyosis. Since glandular components are another important component of adenomyotic foci, further studies are required to understand the entire pathogenesis of adenomyosis. We leave these problems for future work.
